# Increasing the Efficacy of Treatment for Socially Anxious Youth Through Theoretically Derived Improvements: A Pilot Study

**DOI:** 10.1007/s10578-022-01351-6

**Published:** 2022-05-04

**Authors:** Lynda H. Leigh, Frances L. Doyle, Jennifer L. Hudson

**Affiliations:** 1 Department of Psychology, Centre for Emotional Health, Macquarie University, Sydney, Australia; 2 MARCS Institute for Brain, Behaviour and Development, School of Psychology, Western Sydney University, Penrith, Australia; 3 Black Dog Institute, University of New South Wales, Sydney, Australia

**Keywords:** Anxiety, Social anxiety, Cognitive behavioral therapy COTR-D-21–00285

## Abstract

Cognitive behavioural therapy is the first line of treatment for social anxiety disorder; however, children with social anxiety disorder do not respond as well to generic cognitive behavioural therapy programs, compared to children with other anxiety disorders. The aim of the study was to provide a preliminary examination of the efficacy and applicability of a new disorder specific intervention for children with social anxiety disorder. Five children aged 7–13 years, with a primary or secondary DSM-5 diagnosis of social anxiety disorder were provided with an adapted version of the Cool Kids anxiety program. Three out of the five children were in remission from social anxiety disorder at the end of the intervention and at 3-month follow-up. Statistically significant improvements were also noted in overall anxiety symptoms and functioning. Preliminary evidence was found for the efficacy of a social anxiety version of the Cool Kids program.

## Introduction

Anxiety disorders are the most prevalent mental disorders in children and adolescents [1]. Of these disorders, social anxiety disorder (SoAD) is one of the most common, with 12-month prevalence estimated at 1.6% in childhood (4–11 years) and 3.4% in adolescents [12–17 years; 2]. SoAD typically onsets in childhood or adolescence [3] and persists into adulthood [4] with significant disruption to daily functioning, peer relationships, and academic achievement [5]. The adverse effects of the disorder are further exacerbated by the high rate of comorbidity with other anxiety disorders, depression, and substance use [5].

Based on evidence from several meta-analyses and systematic reviews [e.g., 6], cognitive behavioural treatments (CBT) are recommended as the first line of treatment for young people presenting with anxiety disorders, such as SoAD. Historically, cognitive behavioural treatment programs for anxiety disorders in children have been transdiagnostic, as they are designed to target the core construct of anxiety that underlies all of the anxiety disorders. In many research treatment protocols for children with anxiety disorders, children receive the same intervention regardless of the type of anxiety disorder [7–11]. That is, a child presenting with SoAD and a child presenting with Generalised Anxiety Disorder (GAD) would receive the same treatment components, albeit tailored to their individual cognitions and feared situations. These generic protocols predominantly include core components such as cognitive restructuring, gradual exposure and various coping or social skills training [6]. When looking at remission rates across the different anxiety disorders, these generic treatment programs have been shown to be efficacious, with 50% of children achieving remission of the primary anxiety disorder immediately after treatment, and 67% achieving remission within 6 months [6]. Yet when looking at remission rates for specific anxiety disorders, a different picture emerges: Children with SoAD show significant improvements following treatment but do not respond as favourably as children with other anxiety disorders. In a large sample of children and adolescents seeking treatment from our clinic, young people with SoAD demonstrated significantly lower remission rates compared to young people with GAD, Obsessive Compulsive Disorder (OCD), Separation Anxiety Disorder (SAD), and Specific Phobias [12]. For example, 22.3% of children with a primary diagnosis of SoAD were likely to still retain their primary diagnosis compared to only 52.7% of children with a primary diagnosis of GAD. This poorer treatment response is also observed when examining change in symptoms from pre- to post-treatment; individuals with SoAD demonstrated less symptom decline than children with other anxiety disorders. These poorer outcomes occurred when SoAD was the most severe diagnosis and also when it was present anywhere in the diagnostic profile. The poorer SoAD response rates have been identified in numerous studies [13–15].

Poorer outcomes for children with SoAD might occur because the treatment strategies used in generic CBT programs, such as gradual exposure and cognitive restructuring, may not sufficiently target the key factors uniquely involved in the maintenance of the disorder. Although aetiological models of SoAD have been proposed [e.g., 16], there are currently no theoretical models describing the factors involved in the maintenance of SoAD in children and young people. In contrast, there are a number of theoretical maintenance models of adult social anxiety disorder that have been extensively evaluated [e.g., 17, 18]. Strong empirical support for these models has led to the development of novel treatment programs that target the unique maintenance processes, such as the individual’s negative view of the self, self-focused attention, overestimation of cost, safety behaviours, and excessive post-event rumination. For example, adult treatment programs for SoAD focus on modifying the individual’s self-focused attention, by teaching the individual to move their attention away from how they believe they are being perceived by others, and to focus on the positive characteristics of the person they are engaging with, or the task they are completing. This type of attention training is not currently a component of generic youth CBT programs. Adult disorder-specific programs also include the use of video feedback, so that the client is able to obtain more realistic information about performance in social situations. Consistent with this idea, adult programs target the individual’s mental representation of themselves, incorporating feedback not only from video footage but also from other people, suggesting this may be more effective than only using cognitive restructuring to alter the client’s self-perception [18].

The reduction of safety behaviours is another key component of adult SoAD CBT programs given individuals with social anxiety engage in specific safety behaviours to minimise the perceived risk of negative evaluation (such as avoiding eye contact). These subtle safety behaviours not only prevent the individual from gathering evidence to disconfirm their fears, but also tend to result in poorer social performance, thus exacerbating and maintaining the individual’s negative self-perception [18]. To combat this, gradual exposure tasks are performed without using the maladaptive behaviours. To some extent, targeting safety behaviours may be included in generic child programs; however, it is not an essential component of therapy. Regardless, the underlying premise for this approach is that the socially anxious individual does not necessarily exhibit a social skills deficit, rather that it is the individual’s anxiety that prevents them from being able to use their skills in anxiety provoking situations.

Another possible explanation for the poorer response rate for children with social anxiety disorder following CBT, is that individuals with SoAD have more difficulties building an alliance with their therapist, due to the impact of their fear of their therapist. The child’s fear and avoidance within the context of therapy may have an impact on the development of a therapeutic alliance and may impact on the degree to which the child engages with the therapy tasks. Consistent with this, a study has shown that children with social anxiety disorder had poorer alliance with their therapist and poor involvement in therapy compared to children with other anxiety disorders [19]. To address this issue, a disorder specific approach might include strategies to reduce the pressure on the child in the initial sessions to reduce anxiety in the context of the therapy sessions.

By targeting the theoretically proposed maintenance mechanisms of the disorder, enhanced outcomes for individuals with SoAD have been observed in comparison to generic CBT [20, 21]. For example, Rapee, Gaston, and Abbott [20]compared standard CBT to an enhanced treatment package that utilised disorder specific strategies (such as attention training and video feedback), and showed that adults were more likely to demonstrate clinically significant change in the enhanced treatment condition compared to standard CBT. This warrants further exploration as a possible enhancement for child and youth programs. To date, there has been limited evaluation of such programs for children and adolescents. Leigh and Clark [22] trialled the adult social anxiety intervention on adolescents in a series of pilot cases with positive effects with all five adolescents achieving remission of the social anxiety disorder at post treatment. In another pilot study, Ingul, Aune, and Nordahl [23] tested the effectiveness of an adapted disorder specific cognitive therapy program in adolescents aged 13–16 years with a primary diagnosis of SoAD. The enhanced program (delivered individually) showed significantly greater changes in social anxiety symptom severity from pre-treatment to post-treatment, and at 12-month follow-up compared to standard CBT (delivered in a group format). In regard to remission, 72.7% of adolescents in the enhanced condition group no longer met diagnostic criteria for SoAD, compared to 53.3% in the standard CBT; however, due to the small sample size the difference in recovery rates was not significant. In the largest comparison of transdiagnostic versus generic programs for social anxiety disorders to date, Spence et al. [24] compared an online treatment that included social anxiety disorder specific components to a standard online CBT program that focused on cognitive restructuring and gradual exposure in a broad age range of children and youth (8–17 years). There was no significant difference between the two conditions, indicating that disorder specific treatments may not lead to enhanced outcomes for socially anxious youth. Several reasons may explain the absence of difference. The treatment was delivered via online methods, with minimal support from therapists, and there was significant attrition in both conditions. Further there were some key disorder specific strategies that were not included in the intervention that are included in adult social anxiety treatments such as video feedback, behavioural experiments, and a focus on safety behaviours.

The aim of the current study was to determine the preliminary efficacy of a SoAD disorder-specific cognitive behavioural treatment in pre-adolescent children using a case study design. The Cool Kids program, a treatment protocol designed for a range of anxiety disorders [25], was modified to target the cognitive and behavioural processes which maintain SoAD. The present study aimed to examine the efficacy of a targeted SoAD treatment program (Cool Kids Social) in a child population. Based on previous findings, it was hypothesised that the Cool Kids Social program would result in remission of SoAD diagnoses as well as all anxiety diagnoses. In addition, we hypothesised reduction of SoAD severity and associated symptomatology across the course of the study. Finally, given the increased focus on therapeutic alliance we hypothesised high ratings of therapeutic alliance during treatment.

## Method

### Participants

Five children aged between 7 and 13 years (3 females, 2 males) with either a primary or secondary diagnosis of SoAD, as per DSM-5 [26]were included in the study. Table 1 displays participant characteristics. In addition, all five participants spoke English at home and came from two-parent families. Three families identified as Oceanian (2 as North-Western European), 2 reported an average Australian weekly income (1 below average, 2 above average) and 3 children attended Catholic schools (1 private school and 1 public). The Anxiety Disorders Interview Schedule for Children and Parents (ADIS-C/P—IV [27]) was used to assess social anxiety and comorbid disorders. Inclusion in the study required a diagnosis of Social Anxiety Disorder, the child to be aged between 7 and 13 years and the child’s parents to read English. Children were excluded if they presented with a major depressive disorder, suicidal ideation and/or self-harm, or a severe behavioural or intellectual disability. Participants already engaged in concurrent psychological therapy were also excluded.

**Table 1:** Participant characteristics

Age	Gender	Diagnosis and CSR	Cognitions	Safety Behaviours
9	F	*Primary:* SoAD (4) *Secondary:* GAD (4)	“I will look silly” “I will sound silly” “I will sound worried” “People will laugh at me”	Talk softly, avoid eye contact when talking to people, only answer questions I am sure I know the answer to, get mum to ask or answer questions for me, ask mum or other people lots of questions, fiddle with my clothes or hands
7	M	*Primary:* SoAD (7) *Secondary:* Specific Phobia (Animal type) (4)	“People will stare at me” “I will look silly” “People will think I’m weird/dumb” “People will not want to listen to me”	Speak softly, avoid eye contact, play with my clothes, only put my hand up in class if I know the answer, only play with certain kids at school
10	M	*Primary:* GAD (5) *Secondary:* SoAD (4)	“Everyone will think I am terrible” “People will laugh at me” “Everyone will stare at me” “People will not want to talk to me”	Asking mum or other people lots of questions, avoid eye contact, speak quietly, say “I don’t know” to avoid answering a question
9	F	*Primary:* SoAD (5)	“I might make a mistake” “Kids won’t like me” “Kids will be mean to me”	Talk very softly, avoid eye contact, play with my clothes, not sit still
13	F	*Primary:* SoAD (7) *Secondary:* GAD (6)	“People will laugh at me” “People will think I am strange” “I will say something wrong” “I will look awkward/nervous”	Avoid eye contact, talk softly/mumble, fidget, have someone else talk or do things for me, only answer questions I’m sure I know the answer to, ask mum or other people lots of questions, talk fast

*CSR* clinician severity rating, *SoAD* social anxiety disorder, *GAD* generalised anxiety disorder

### Case Summaries

Names have been changed in each case.

*Roxy* (Participant 1) was a 9-year-old girl who was referred to the program because of persistent worries about being embarrassed in front of her classmates, which impacted her engagement in class. At assessment it was established that she consistently avoided answering questions in class, giving speeches, and asking for help. In addition, Roxy avoided talking to new or unfamiliar people and was often worried that she would embarrass herself. During the initial sessions Roxy avoided eye contact, frequently said “I don’t know”, fidgeted and moved around on her chair, and often looked to her mother to answer questions. Roxy met criteria for Social Anxiety Disorder (Clinical Severity Rating CSR 4), and an additional diagnosis of Generalised Anxiety Disorder (CSR 4).

*Mason* (Participant 2) was a 7-year-old boy who was referred to the program because of his continued avoidance of activities and social interactions at school and in other situations. Mason’s school teacher had noticed his anxiety and suggested further investigation. Assessment revealed that Mason avoided asking and answering questions in class, musical performances, starting or joining in on conversations, speaking to people in general, and going to parties or other social events. In the initial sessions, Mason was very quiet and spoke softly when answering questions. Mason was also observed to fidget extensively when talking about his worries. Mason met criteria for Social Anxiety Disorder (CSR 7) and an additional Specific Phobia diagnosis (Animal type).

*Nathan* (Participant 3) was a 10-year-old boy whose school teacher recommended assessment due to his anxiety, which was interfering with school performances and friendships. During assessment, it was established that Nathan experienced anxiety in many social situations, but particularly when speaking to adults, starting or joining in on a conversation, answering questions in class, and doing an oral presentation. During the initial sessions, Nathan would regularly say “I don’t know” and often became visibly upset when asked about his worries, particularly in the presence of his parents. Nathan found it difficult to disclose his worried thoughts when completing detective thinking; however, he found it easier if he was asked to provide thoughts for a hypothetical person (for example, the character Indy in the manual). Nathan met criteria for Generalised Anxiety Disorder (CSR 5) and Social Anxiety Disorder (CSR 4).

*Chelsea* (Participant 4) was a 9-year-old girl who was referred to the program by her parents who were concerned about her ongoing difficulties with peer interactions and standing up for herself. Initial assessment indicated that Chelsea endures distress and avoids starting or joining in on a conversation, asking questions in class, standing up for herself, and performing in front of people. In the early sessions, Chelsea spoke very softly and did not provide information spontaneously. When talking about her worries, Chelsea became visibly upset at times. Chelsea met criteria for Social Anxiety Disorder (CSR 5).

*Samantha* (Participant 5) is a 13-year-old girl who was referred to the program by her paediatrician for concerns regarding social anxiety. It was noted at the assessment that Samantha has a history of anorexia, for which she had previously been treated. Assessment revealed that Samantha avoids or endures distress in almost all social situations. In particular, she avoids: speaking to unfamiliar people, starting or joining in on a conversation, attending school camp, and participating in sports class or athletic performances. Samantha’s anxiety interferes with her ability to form friendships and to be independent. For example, Samantha avoids buying items in a shop or ordering food and will often ask her younger brother to do this for her. In the initial sessions, Samantha spoke openly about her worries but avoided any eye contact, often facing her body away from the therapist. Samantha met criteria for Social Anxiety Disorder (CSR 7) as well as an additional diagnosis of Generalised Anxiety Disorder.

### Measures

Pre- and post-treatment assessments were multi-informant and multi-method. Mid-treatment assessments were questionnaires completed by the parent, child, and therapist.

#### Outcome Measures

As described above, the ADIS-IV-C/P [27], a clinician administered semi-structured diagnostic interview was used to assess the presence and severity of anxiety disorders.

The Liebowitz Social Anxiety Scale for Children and Adolescents (LSAS-CA) [28] is a 24-item clinician administered scale that assesses both anxiety and avoidance in social interactions and performance situations. Children and adolescents used a Likert type scale to rate anxiety either 0 (*none*), 1 (*mild*), 2 (*moderate*), or 3 (*severe*). Avoidance was rated as 0 (*never*), 1 (*sometimes)*, 2 (*often*), or 3 (*usually*). Situations assessed for anxiety and avoidance include “talking with other kids you don’t know well”, “looking at people you don’t know well in the eyes”, and “giving a verbal report or presentation in class”. The LSAS-CA has shown good to excellent internal consistency (α = 0.83–0.97) and test–retest reliability (α = 0.89–0.94) [28]. The LSAS-CA has also been found to have strong correlation with other measures of social anxiety [28].

The Spence Children’s Anxiety Scale (SCAS) [29] was used as a general measure of anxiety as well as an additional measure of social anxiety. The SCAS consists of a 44-item self-report measure (of which 38 items are scored) and a 38-item parent report measure [30] that assesses six domains of anxiety, including social phobia. The social phobia subscale was completed by both the child and parent at pre-treatment, mid-treatment, and post-treatment. Items are score using a Likert scale ranging from 0 (*never*) to 3 (*always*). Example items are “I worry what other people think of me” and “I feel afraid if I have to talk in front of my class”. The social phobia subscale has been found to have adequate internal consistency (α = 0.73–0.75) and test–retest reliability (α = 0.75) [31].

The Children’s Global Assessment Scale (CGAS) [32] is a clinician rated measure of functioning in children and adolescents. Functioning was assessed on a scale from 1 *(needs constant supervision*) to 100 (*superior functioning*). To rate the child or adolescent, clinicians refer to a glossary that defines the meaning of the points in the scale. Psychometric properties of the CGAS are good, with test–retest interrater reliability of α = 0.83 [33].

#### Depressive Symptoms

Depressive symptoms were assessed using the child and parent report versions of the Short Mood and Feelings Questionnaire (SMFQ) [34]. The SMFQ consists of 13-items rated on a three-point Likert scale of 0 (*not true*), 1 (*sometimes*), and 2 (*true*). Symptoms were reported for the previous two weeks. Example items include “I felt miserable or unhappy” and “I was very restless”. The SMFQ has good reliability (α = 0.85-0.87) [34]. Studies have indicated that the parent report predicts depression better than the child report; however, predictive ability was increased when both the parent and child versions were used together [34].

#### Therapeutic Alliance

The Therapeutic Alliance Scale for Children, Revised (TASC-r) [35, 36] assesses therapeutic alliance through the use of positive and negative statements such as “I felt like my therapist was on my side and tried to help me” and “I would rather have not worked on my problems with my therapist”. The TASC-r is a 12-item self-report measure, with parallel report versions available for the parent and therapist. Items are scored using a Likert scale ranging from 1 (*not true*) to 4 (*very much true*), with negative items reverse coded. Internal consistency ranges from good to excellent, with Cronbach alphas of α = 0.88–0.92 (child report), α = 0.94–0.96 (therapist report) (Creed & Kendall, 2005), and α = 0.85–0.88 (parent report) [37].

#### Automatic Thoughts

Cognitions were assessed at pre-treatment, mid-treatment, and post-treatment using the Children’s Automatic Thoughts Scale (CATS) [38]. The CATS is a self-report measure consisting of 40 negative self-statements that are associated with internalising and externalising problems. It comprises four subscales: physical threat (e.g., “I am going to have an accident”), social threat (e.g., “kids will think I’m stupid”), personal failure (e.g., “I can’t do anything right”), and hostility (“people always try to get me into trouble”). Children and adolescents rate the frequency of each thought over the past week, using a 5-point Likert scale that ranges from 0 (*not at all*) to 4 (*all the time*). The total score for the scale was used for this study. The scale has demonstrated excellent overall internal consistency (α = 0.95) and good to excellent consistency for each of the subscales (α = 0.82–0.92) [39].

#### Reliable Change in Social Anxiety Symptoms

LSAS-CA scores and SCAS-Social scores were assessed using Jacobson and Truax’s [40] criteria for reliable and clinically significant change. For the LSAS-CA reliable change was calculated using the test–retest coefficient for the total score (α = 0.94) and data from a non-psychiatric population with a standard deviation of 5.8; Reliable Change Index = 3.94 [28]. For SCAS-C-Social, the 6-month test re-test coefficient (α = 0.57) and standard deviation of 3.70; Reliable Change Index = 6.26 (Spence, 1998). For the SCAS-P-Scoial, the internal consistency (α = 0.74) and standard deviation of 3.10.; Reliable Change Index = 4.39 (Nauta et al., 2004).

### Procedure

This study was approved by the Human Research Ethics Committee of Macquarie University. Parents self-referred their child to the Centre for Emotional Health, Macquarie University, Sydney. Telephone intake was conducted with the primary caregiver to determine if the child has symptoms of anxiety and is not immediately identifiable as meeting exclusion criteria. Participants who were suitable and who agreed to participate in the study were scheduled for a diagnostic interview to assess the child’s anxiety and were requested via email to complete a questionnaire pack (one for the child and one for the primary caregiver). At the diagnostic interview a clinician administered the ADIS—C/P. Children who met criteria for inclusion were then offered a place in the present study and emailed a copy of the Participant Information Consent Form. Parents provided written consent and children provided verbal consent.

During therapy, the TASC was administered to parents and children at sessions 1 6 and 10. At session 6, the child and parents were also asked to complete the SCAS and the CATS. At the end of the treatment program, and again at 3 months after the completion of the treatment, children and parents were interviewed using the ADIS-C/P (second author) who was unaware of the severity of the initial diagnoses and not aware of the details of the treatment program or process. Symptom measures were also completed at this time. All families completed the treatment and all participated in the post assessment. One family was unable to attend the follow-up assessment.

#### Therapist and Supervisor

Sessions were delivered by either LL, a postgraduate student in clinical psychology, or JH, an experienced psychologist. Both therapists had previously been trained in the original Cool Kids program and had experience delivering the program. JH, lead author of the Cool Kids Social program, provided LL with weekly supervision.

#### Treatment

The Cool Kids Social program [41] is a manual based cognitive behavioural treatment designed to be delivered in 10 individual 1-h sessions. All sessions involved both the young person and at least one parent, except for session five which was a parent-only session. Appointments were held weekly for sessions one to eight, and then fortnightly for the remaining two sessions to allow for increased practice.

The original Cool Kids program includes the following components: psychoeducation about anxiety, identifying thoughts and feelings, cognitive restructuring (detective thinking), parenting strategies, and exposure stepladders. Optional components include dealing with bullying, and other coping skills such as problem solving. The Cool Kids Social program differed from the standard program in the following ways: allocation of additional time for rapport building, examples specific to social anxiety, inclusion of attention retraining, inclusion of detective thinking for post-event processing, and inclusion of performance feedback (including video feedback) and a focus on reducing safety behaviours. In addition, exposure tasks were presented as behavioural experiments. A summary of the session content can be found in Table 2. The following is an outline of the content.

**Table 2:** Summary of treatment components

Session	Content
1	Rapport building, psychoeducation, worry scale, goal setting
2	Rapport building, fears and worries list, linking thoughts and feelings, attention training (breathing exercise)
3	Detective thinking, choosing rewards
4	Detective thinking (so what question), behavioural experiment list, in-session exposure
5	*Parent session*. Review of progress, patterns of parenting, strategies for parenting an anxious child
6	Safety behaviours, experiment list (safety behaviour experiments), attention training (self-focused vs task-focused attention)
7	Video feedback experiment (accurate self-perception), experiment list (feedback experiments), in-session exposure
8	Detective thinking (post-event processing), extra challenge experiment list (cost exposure), in-session cost exposure
9	In-session exposure, review of experiment list, troubleshooting experiments (with parents), optional module (teasing and bullying)
10	Review of goals and progress, preparing for setbacks, big challenge planning

In session one, a considerable amount of time was spent building rapport with the young person. As talking to the therapist is likely to be a significant source of anxiety for a child with SoAD, content that required substantial input or communication from the participant was kept to a minimum to avoid undue pressure. Strategies included using an ice breaker game, using closed questions, and allowing the child to choose to write down or draw responses instead of answering verbally (if this was a preferred method). In addition, a character named Indy (who can be any gender) is introduced. Indy is a child with social anxiety and the use of this character throughout the program is intended to take the focus off the client and to normalise the thoughts, feelings, and behaviours that are discussed. Session one included psychoeducation about social anxiety, the worry scale (a distress scale ranging from 0 “very relaxed” to 10 “extremely worried”) was introduced, and children and parents created goals.

Session two started with more rapport building. The children then created a “fears and worries” list (a list of their worries separated into three levels: *makes me a little worried*, *hard to do*, and *really hard to do*) and practised linking thoughts and feelings. Attention training was introduced through the inclusion of a breathing exercise, designed to help the child to improve their ability to focus on a specified stimulus. The rationale for the attention training exercise was that attention is like a muscle, in that it needs training to get stronger. By practising the breathing exercise, the child strengthened their attention. The child was encouraged to practice the breathing exercise at home each week, along with practicing linking thoughts and feelings.

Session three introduced cognitive restructuring through use of “detective thinking”. This was practiced in session and assigned as a homework task, along with attention training. The young person and their caregiver were also encouraged to choose potential rewards for exposure tasks before the following session.

In session four, detective thinking was extended to encourage the child to examine and challenge the costs associated with their feared situation. This process aimed to help the child recognise that even if the worst happened, they would be able to cope. Behavioural experiments were also introduced. Behavioural experiments required the child to face feared situations so that they can gather information about the validity of their thoughts and beliefs. The child worked together with their caregiver and therapist to create an experiment list and design their first experiments. The experiment list was created by brainstorming ways of testing out the child’s worried thoughts and then grouping them into small, medium, and hard (based on worry rating). An in-session experiment was conducted with the child to demonstrate the process and how to complete the experiment worksheet. Experiments and attention training were set as homework.

Session five was a parent-only session and focused on effective parenting strategies for parenting a child with anxiety. In particular, parents were asked to identify any patterns of behaviour that may be maintaining their child’s anxiety (such as allowing avoidance or providing excessive reassurance). Parents were encouraged to choose one or two behaviours to work on over the week.

In session six, safety behaviours were explained and the child identifies the safety behaviours they use when feeling anxious. An in-session experiment was conducted twice, first with the child using their safety behaviours and then without using them. After each experiment, the child was asked to rate their worry and performance. Afterwards, the child compared the ratings. This exercise aimed to highlight to the young person that although they may feel less anxious the first time, their performance was likely to be better the second time. The role of safety behaviours in maintaining anxiety was also discussed. Attention training was expanded upon and role-plays used to highlight the difference between self-focused attention and task-focused attention. In the first role-play, the child was encouraged to focus their attention on themselves and how they were being perceived by the other person. In the second role-play, they were encouraged to focus on the task (a conversation or listening activity). The child was then asked to engage in a weekly homework task where they practiced using task-focused attention in various social situations.

Session seven focuses on helping the child gain an accurate self-perception. The child was asked to talk for a short amount of time on any topic whilst being video recorded. Before the speech, the child rated how anxious they felt and how anxious they thought they would look. They complete these ratings again after the speech. In addition, after the speech, the child listed the ways they thought their anxiety would be noticed (e.g., blushing) and then rated how noticeable these signs were during the speech. The child was then asked to watch the video objectively before rating their performance again. The aim was for the children to recognise they look less anxious than they think. Building on this exercise, behavioural experiments that include ways of obtaining feedback were created for the child to practise during the next week, along with their other experiments and attention re-training. An in-session experiment was also conducted.

In session eight, post-event processing was explained and the child generated a list of situations in which they may be able to use detective thinking to challenge worries that occur after an event. A detective thinking sheet was completed for one of the identified situations. The child was then introduced to the idea of cost-exposure and “extra challenge” experiments were created. The aim was for the young person to learn that the consequences of something “going wrong” were not as bad as they expected and to realise that they can cope with any consequences that arise. An extra challenge experiment (an exposure to cost) was conducted in session. Homework consisted of extra challenge experiments, other behavioural experiments, detective thinking for post-event worries, and attention retraining.

For the child, session nine consisted of several in-session behavioural experiments. A review of the child’s fear and worries list was also included, and new experiments were designed for any fears or worries that have not yet been challenged. A short time was also spent alone with the parent, reviewing progress and troubleshooting any problems with completing experiments. An optional bullying module was included if the young person was having significant issues with bullying. For this component, an action plan was created for situations in which the child was typically bullied. Role plays were used to practice interactive components of the action plan.

Session ten focused on reviewing the progress the young person had made during the program. Any experiments or goals that were still left were planned. The possibility of future setbacks was discussed, including ways for the child and parent to manage setbacks when they occur. The child was also encouraged to think of a “big challenge” that they can work towards to ensure continued practice. Treatment notes are available in the Appendix.

## Results

### Clinical Outcomes

**Fig. 1:**
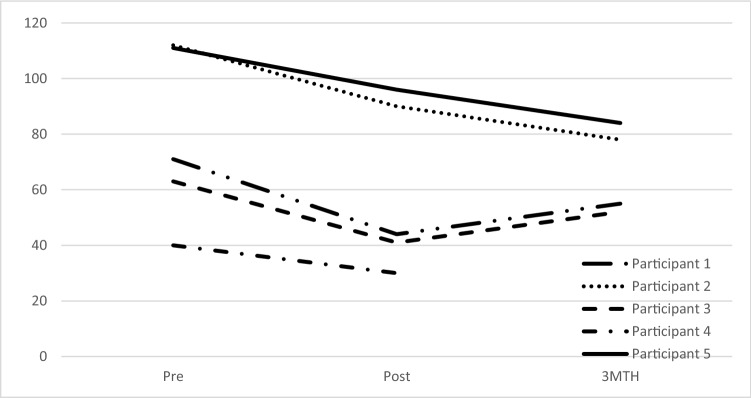
Pretreatment, posttreatment and 3 month follow-up LSAS-CA scores

Table 3 presents the clinical outcome measures. At the end of the intervention and at three-month follow-up three out of the five participants no longer had a diagnosis of SoAD or any anxiety diagnosis. The remaining two participants had 2-point reductions in their CSR at post-treatment and 3-point reduction at 3-month follow-up. Figure 1 depicts the changes in LSAS-CA scores from pre-treatment to post-treatment and 3-month follow-up. All participants met criteria for reliable and significant change on the LSAS-CA at post and follow-up and mean difference of 19.2 at post and 12.1 at follow-up. Changes in SCAS social phobia subscale scores (parent report) were observed, with a mean decrease of 2.6 at mid, 3.8 at post, and 4.2 at follow-up. Reliable change index was reached for three participants at the follow-up point (assuming post data for Participant 4 is carried forward). Changes in the SCAS social phobia subscales (child report) were observed with a mean decrease of 1 point at mid, 3 at post and 4 at follow-up. Three of the participants report a score of zero at the end of treatment. The reliable change index was reached for one participant but this is unreliable given that three of the pre-treatment scores were below the normative mean. Improvements in scores on the CGAS, CATS, SCAS-Total wand SMFQ-parent report observed between pre-treatment, post-treatment, and follow-up. No improvement in SMFQ child report was observed across time.

**Table 3:** Clinician, Parent and child reported outcome measures across time

		Participant					
		1	2	3	4	5	Mean (SD)
ADIS-C/P	Post	Yes	No	Yes	Yes	No	
Remission- Social Anxiety	3 M	Yes	No	Yes	–	No	
Remission- All Anxiety	Post	Yes	No^a^	Yes	Yes	No^b^	
	3 M	Yes	No^a^	Yes	–	No^b^	
Social Anxiety CSR	Pre	4	7	4	5	7	5.4 (1.5)
	Post	3	5	3	3	5	3.8 (1.1)
	3 M	2	4	3	–	4	3.23 (.96)
CGAS	Pre	58	45	53	56	50	52.4 (5.1)
	Post	70	47	69	79	53	63.6 (13.2)
	3 M	90	68	79	–	68	76.3 (10.53)
LSAS-CA (Total Score)	Pre	71	112	63	40	111	79.4 (31.4)
	Post	44	90	41	30	96	60.2 (30.5)
	3 M	55	78	52	–	84	67.3 (16.11)
SCAS Social Scale (Parent)	Pre	9	11	13	9	9	10.2 (1.8)
	Mid	7	10	6	6	9	7.6 (1.8)
	Post	6	10	5	3	10	6.8 (3.1)
	3 M	2	9	7	–	6	6 (2.9)
SCAS Social Scale (Child)	Pre	11	3	5	3	12	6.8 (4.4)
	Mid	6	9	2	0	12	5.8 (4.9)
	Post	1	5	4	0	9	3.8 (3.6)
	3 M	0	0	3	–	8	2.8 (3.8)
SCAS Total (Parent)	Pre	32	33	38	23	21	29.4 (7.2)
	Post	17	19	20	13	18	17.4 (2.7)
	3 M	8	13	20	–	9	12.5 (5.5)
SCAS Total (Child)	Pre	55	19	21	19	41	31 (16.3)
	Post	12	34	14	12	34	21.2 (11.7)
	3 M	11	19	14	–	33	19.25(9.7)
SMFQ (Parent)	Pre	0	0	4	10	0	2.8(4.4)
	Post	0	0	4	2	0	1.2 (1.8)
	3 M	0	0	0	–	0	0 (0)
SMFQ (Child)	Pre	0	1	4	4	2	2.2 (1.8)
	Post	0	0	2	5	2	1.8 (2.0)
	3 M	6	0	2	-	2	2.5 (2.5)
CATS (Total)	Pre	0	1	2	5	23	6.2 (9.6)
	Mid	2	1	1	7	–	2.8 (2.9)
	Post	0	0	0	12	25	7.4 (11.1)

*ADIS-C/P social anxiety CSR* Anxiety Disorders Interview Schedule for Children and Parents Clinician Severity Rating, *CGAS* Children’s Global Assessment Scale, *LSAS-CA* Liebowitz Social Anxiety Scale for Children and Adolescents, *SCAS* Spence Children’s Anxiety Scale *SMFQ* Short Mood and Feelings Questionnaire, *CATS* Children’s Automatic Thoughts Scale

^a^Participant 2 no longer met criteria for Specific Phobia at post or 3 M

^b^Participant 5 also met criteria for GAD at post but not at follow-up

### Therapeutic Alliance

Table 4 displays therapeutic alliance scores measured at weeks 1, 6, and 10. Mean therapeutic alliance scores were noted to increase over the course of the intervention for clinician, parent and child report.

**Table 4:** Scores and means for therapeutic alliance

Participant							
		1	2	3	4	5	Mean (SD)
TASC-r (Parent)	W1	40	46	43	42	41	42.4 (2.3)
	W6	40	46	40	43	45	42.8 (2.8)
	W10	43	47	40	48	47	45 (3.4)
TASC-r (Child)	W1	44	46	35	42	39	41.2 (4.3)
	W6	42	45	39	45	45	43.2 (2.7)
	W10	45	43	40	47	46	44.2 (2.8)
TASC-r (Therapist)	W1	25	34	36	36	40	34.2 (5.6)
	W6	34	36	35	35	44	36.8 (4.1)
	W10	35	37	37	38	46	38.6 (4.3)

*TASC-r* Therapeutic Alliance Scale for Children, Revised Shirk et al. [35], *W1* Week 1, *W6* Week 6, *W10* Week 10

## Discussion

This pilot study provides initial support for the efficacy of the Cool Kids Social program [41] for the treatment of Social Anxiety Disorder in children. The Cool Kids Social program is a cognitive behavioral treatment program designed to address the theoretically proposed mechanisms involved in the maintenance of social anxiety. These strategies include attention training to reduce self-focused attention, video feedback to reduce safety behaviors, and behavioral experiments with a focus on exposure to cost. The program also pays particular attention to building the therapeutic alliance and reducing social threat within the therapy session. As hypothesized, all five children experienced a reduction in the severity of their social anxiety, with three out of the five children being free of their SoAD diagnosis by the end of the intervention. Child and parent reported anxiety symptoms were also lower at the end of treatment and at three months following the intervention. The rate of remission of social anxiety diagnoses observed in this study was 60% at both post-treatment and follow-up. This is notably higher than remission rates observed in children with SoAD after completing the standard Cool Kids program (22.3% Post; 30.7% [12]). Although treatment focused on social anxiety disorder, all comorbid anxiety disorders had remitted by the end of the follow-up period. Strong therapeutic alliance that increased over time was also observed across all reported. In combination, these results provide preliminary support for the use of this program with children presenting with social anxiety disorder.

Although these results are promising, they are not as strong as those reported in a similar case series involving a sample of adolescents with social anxiety disorder [22]. Notably, our remission rate did not match the 100% remission rate observed by Leigh and Clark, and we also witnessed a lower decrease in social anxiety symptoms. Several methodological differences between these studies may explain the discrepancies in results. First, the intervention period was much longer in the study by Leigh and Clark [22], with more than double the amount of hours spent treating the clients than the present study. There has been some evidence that more exposure sessions are associated with improved outcomes in social anxiety [42], suggesting that interventions with more exposure sessions may be advantageous for these individuals. Longer interventions may also allow more time for rapport building, thus promoting a stronger therapeutic alliance which may then improve treatment outcomes [43]. Further, the present study examined a significantly younger population than Leigh and Clark (2016). Although an individual patient data meta-analysis found that age does not impact CBT treatment outcomes [44], it is possible that the lower level of cognitive maturity in our sample may have made it more difficult for the participants to grasp the concepts involved in the cognitive components of the protocol. As an example, in session seven of our program, the aim of the video-feedback exercise was for the child to have an “a-ha” moment (i.e. to recognise that they do not look as anxious as they imagine). In contrast to what we expected, therapists reported there was little discrepancy between the child’s ratings of their performance before and after the videoing. As such, the children were unable to grasp the concept of this exercise. While all participants in our study improved significantly over the course of treatment, it is unclear whether cognitive maturity negatively impacted outcomes. This could be explored in further studies by using the adapted program with adolescents.

Results of the present study provide initial evidence for the efficacy of the Cool Kids Social program in treating children with SoAD. As this is a preliminary investigation conducted with a small number of children, this intervention needs to be evaluated in a randomised controlled clinical trial. Importantly, we need to know whether it is more efficacious than existing transdiagnostic treatments for children with social anxiety. As the treatment program is designed to target a number of mechanisms proposed to maintain social anxiety disorder in children [17, 18, 45], future trials would benefit from measuring changes in these proposed constructs, such as self-focused attention, post event rumination, and safety behaviours.

### Summary

Numerous studies have identified that children with social anxiety disorder respond less favourably to generic cognitive behavioural treatment programs compared to children with other anxiety disorders. Emerging evidence suggests that disorder specific treatments may result in more favourable outcomes for these children. To address this, the current study used a case series design to evaluate the efficacy of a disorder specific treatment for children with social anxiety disorder. The results of the current case series provide preliminary evidence of the efficacy of the Cool Kids Social program in reducing anxiety in children presenting with social anxiety disorder. Our results are promising and our findings contribute to the emerging research on disorder specific interventions for children and adolescents with SoAD.

## Appendix: Treatment Notes

*Roxy* (Participant 1): An important element of the program for Roxy was cost exposure, which allowed her to gather more realistic information about the situation and to learn that she could cope with any negative experiences. Cost exposure experiments included saying words wrong when reading out loud to an adult and wearing brightly coloured dress-up clothes around the lobby. Another important aspect of treatment for Roxy was reducing her safety behaviours. It became apparent during the sessions that Roxy would stay silent and wait for her mother to speak on her behalf. This was a point of discussion in the parent session and in the remaining sessions Roxy gained confidence speaking for herself in-session. For the experiments in session nine, Roxy was able to introduce herself to an unknown staff member and have a short conversation about likes and dislikes. During the conversation, she was able to speak confidently and maintain appropriate eye contact. By the end of the intervention, Roxy was able to: put her hand up in class and guess the answer to a question, make eye contact with new and unfamiliar people, have a short conversation with an unknown adult, and wear bright party accessories out in public.

*Mason* (Participant 2) Mason found it difficult to identify and articulate his worried thoughts in-session but could complete detective thinking verbally with his mother outside of the session. Behavioural experiments were particularly useful for Mason and his parents ensured that many of these were implemented outside of the session. Experiments included kicking his soccer ball over the fence and having to go and ask the neighbour for it back, and walking down the road near his house wearing dress-up clothes. Mason was able to learn that situations were not as scary as he initially thought. At the completion of intervention, he was able to put his hand up in class more often to answer questions and his teacher had indicated that he was talking to her and other children in the class more. In addition, Mason was able to independently buy items from shops (including the school canteen), could talk in front of his class with less worry, and was better at asking people questions or for help.

*Nathan* (Participant 3) The use of the “so what” question in detective thinking was helpful for Nathan. He was often able to recognise that even though he worried, he would be able to cope with the worst outcome. The in-session extra challenge experiments were also important for Nathan as he was able to successfully face his fears in high risk situations which then encouraged him to try more cost exposure experiments outside of the therapy session. Cost exposure experiments including singing while standing on the stairs in the lobby and wearing a brightly coloured party wig and oversized party glasses while walking around a busy library. At the completion of the intervention, Nathan was more confident when buying items from a shop or ordering something at a café/restaurant. He was able to have longer conversations with adults (including asking for help or information), and his level of reassurance seeking had reduced. Nathan’s parents also reported that he was able to talk about his worries more openly and was happier at school.

*Chelsea* (Participant 4) Chelsea found it very difficult to disclose her predictions about situations and so she struggled to complete the detective thinking sheets with her parents at home and in session. It was also very difficult to develop behavioural experiments as she would not provide any negative cognitions. It became clear that Chelsea was highly anxious about disclosing her thoughts when she was the centre of attention. When she was left to complete the detective thinking on her own when there was no pressure (e.g., when the therapist and parents were not present in the room with her) she was able to write down clear and concise predictions about what she was concerned would happen in the situation. She was also able to generate more accurate evidence and develop a more realistic thought to use. This was very useful to discover as it allowed more precise tailoring of behavioural experiments. During the session on safety behaviours, Chelsea during the second in-session experiment was able to use a louder voice and use eye contact very competently. Both Chelsea and her parents reported an improvement in her perceived performance. The video-feedback session also proved useful for identifying an additional safety behaviour (playing with her clothes). In session nine, the additional bullying module was delivered as Chelsea and her parents had reported that she had been bullied at school and had been unable to stand up for herself. In session, Chelsea practiced some good comebacks and practiced without using her safety behaviours. Cost exposures included singing loudly in the building lobby and deliberately making a mistake in a soccer game. By the end of the intervention period, Chelsea was able to join two sporting clubs/teams, was talking to her school teacher more, using a louder voice when speaking, was standing up to her siblings more, and was better able to talk about her feelings.

*Samantha* (Participant 5) Samantha commented that the behavioural experiments were the most useful component of the program. These experiments included independently buying items from a shop, asking for directions, and making an odd sound in front of her peers. Given the extent that Samantha avoided social situations prior to treatment, the behavioural experiments were crucial for Samantha to gather more realistic information about her fears and to learn that she could stay in situations despite feeling very anxious. A particularly important in-session experiment for Samantha was playing a staring game with the therapist in an attempt to reduce her safety behaviour of avoiding eye contact. In addition, the importance of attention retraining became clearer to Samantha during this experiment, as she realised that it was easier to maintain eye contact if she focused on noticing the facial features of the therapist rather than thinking about how awkward she felt or how weird she thought she looked. By the end of the intervention Samantha was able to: make eye contact with new people, buy something from a shop or café by herself, approach people to ask a question, and talk to unfamiliar classmates.
